# The development of Breast Apocrine Carcinoma (BAC) score that distinguishes breast and cutaneous apocrine carcinomas

**DOI:** 10.21203/rs.3.rs-9642856/v1

**Published:** 2026-05-26

**Authors:** Kei Kawashima, Akimitsu Yamada, Eleanor A. Fallon, Ryo Fujita, Mahato Sasamoto, Masanori Oshi, Kazutaka Narui, Hideyuki Ishikawa, Jotaro Harada, Yukie Yamaguchi, Satoshi Fujii, Takashi Ishikawa, Itaru Endo, Kazuaki Takabe

**Affiliations:** Roswell Park Comprehensive Cancer Center; Yokohama City University Graduate School of Medicine; Roswell Park Comprehensive Cancer Center; Yokohama City University Graduate School of Medicine; Yokohama City University Graduate School of Medicine; Yokohama City University Graduate School of Medicine; Yokohama City University Medical Center; Yokohama City University Graduate School of Medicine; Yokohama City University Graduate School of Medicine; Yokohama City University Graduate School of Medicine; Yokohama City University Graduate School of Medicine; Yokohama City University Graduate School of Medicine; Yokohama City University Graduate School of Medicine; Roswell Park Comprehensive Cancer Center

**Keywords:** Breast apocrine carcinoma, cutaneous apocrine carcinoma, differentiation, transcriptome, discrimination score

## Abstract

**Background:**

Apocrine carcinoma (AC) is a rare cancer that arises either from cutaneous apocrine sweat glands (cutaneous AC) or as breast carcinoma with apocrine differentiation (breast AC). Differentiation between them is clinically critical because their management and outcomes differ significantly: breast AC is treated according to established breast cancer therapies, whereas cutaneous AC lacks standardized systemic treatment and often carries a poorer prognosis. A diagnostic challenge arises when AC occurs in the axilla, where both entities may arise, given their highly similar histology.

**Methods:**

In this study, we performed a retrospective multi-cohort transcriptomic analysis integrating institutional, multi-institutional, and publicly available datasets to characterize the molecular differences between breast and cutaneous AC, and developed a transcriptomic classifier using differentially expressed genes and a LASSO logistic regression model for tissue-of-origin classification.

**Results:**

Transcriptomic profiling revealed that breast ACs exhibited higher steroid hormone–related signaling and lower proliferative activity compared with cutaneous ACs, while immune-related features were largely comparable between the two tumor types. These findings suggest that, despite shared apocrine morphology, breast and cutaneous ACs retain distinct biological characteristics reflective of their tissue of origin. Building on this, we further developed a transcriptomic classifier, the Breast Apocrine Carcinoma (BAC) score, which accurately distinguished breast and cutaneous ACs, suggesting that these tumors retain distinct tissue-of-origin transcriptional signatures despite shared apocrine differentiation. Furthermore, application of the BAC score to tumors with unknown primary origin in the axillary region enabled estimation of tissue origin, highlighting its potential clinical utility in diagnostically challenging cases.

**Conclusion:**

Breast and cutaneous ACs exhibit distinct biological features despite shared apocrine morphology. The BAC score accurately distinguished tumors of breast and cutaneous origin, including within AC cohorts, highlighting the potential utility of transcriptomic profiling for improving their classification and diagnostic evaluation.

## Introduction

Apocrine glands are specialized sweat glands in the skin that secrete lipid-rich sweat and are predominantly distributed in the axilla [1]. Tumors derived from these glands exhibit apocrine differentiation and are collectively referred to as cutaneous apocrine carcinomas (AC) [2], which is a rare adnexal malignancy of the skin. Incidence of cutaneous AC ranges from approximately 0.0049 to 0.23 per 100,000 persons per year across different populations and databases, and no significant temporal change observed in recent epidemiologic analyses [2]^,^[3]. Interestingly, AC can also occur in the breast. The mammary glands in breast and apocrine glands share common embryological origins [4]^,^[5]^,^[6], and apocrine differentiation is commonly observed in breast tissue [7]. As a matter of fact, autopsy studies have shown that 50–85% of normal breast tissue exhibits some degree of apocrine morphology [8]^,^[9]^,^ [10]. When apocrine morphology occupies the majority of cancer cells, the tumor is classified as breast AC [11], which accounts for approximately 0.4–4% of all breast cancers [12]^,^[13].

Differentiation between breast and cutaneous AC is clinically critical, as treatment strategies and prognoses differ substantially. Breast AC is generally managed according to therapeutic approaches for invasive breast carcinoma of no special type (NST), with therapeutic decisions being guided by tumor subtype [12]. Breast AC has been reported to show relatively favorable clinical outcomes compared with non-apocrine breast cancers, particularly within triple-negative breast cancer (TNBC) [13]^,^[14]^,^[15], which otherwise is considered the most aggressive subtype with the worst outcomes. Building upon this, we recently reported that the presence of apocrine morphology in breast cancer is associated with distinct estrogen receptor (ER)-dependent biological and clinical features (Kawashima et al., manuscript under review [16]). In contrast, cutaneous AC is primarily treated with surgical excision, and there is currently no established systemic therapy [17]. The prognosis of cutaneous AC has been reported to be poor, with median survival ranging from approximately 33 to 55 months depending on nodal status [18].

Although critically important, distinguishing breast and cutaneous ACs axilla has long been a clinical challenge [18] particularly in the axilla because both subtypes can occur in this anatomic region [2, 19]^,^ [20]^,^[21]. Ectopic breast tissue most commonly occurs in the axilla [22] and present in approximately 1–6% of the population [23]^,^[24]. Although rare, this tissue can give rise to ectopic breast cancer, which accounts for approximately 0.3–0.6% of all breast cancers [20] [21]. Conversely, cutaneous ACs frequently occur in the axilla as it is rich in apocrine sweat glands [19] [2]. Histologically, breast and cutaneous AC share highly similar morphological features [18]. Immunohistochemical markers commonly used in clinical practice, including mammaglobin and gross cystic disease fluid protein (GCDFP), also show substantial overlap between the two entities, reflecting shared apocrine differentiation [18]^,^[6]. To this end, there is an unmet need in the means to differentiate breast AC to cutaneous AC for the application of appropriate treatment to the right patients.

To date, comprehensive molecular comparisons between breast and cutaneous ACs have not been systematically performed. In particular, transcriptomic differences between the two entities remain largely understudied. Given this unmet clinical need, we conducted a retrospective multi-cohort study to comprehensively characterize the clinical and molecular distinctions between cutaneous and breast ACs. Furthermore, we developed a novel transcriptomic scoring system, the Breast Apocrine Carcinoma (BAC) score, to facilitate their differentiation, which may serve as a potential tool to distinguish cutaneous and breast ACs.

## Material and Methods

### Study design and patient cohort

This retrospective multi-cohort study included a multi-institutional cohort from the Oncology Research Information Exchange Network (ORIEN) and an institutional cohort from Yokohama City University (YCU) hospital, and publicly available transcriptomic datasets. The ORIEN cohort included patients diagnosed with breast cancer (n = 1797) or skin cancer (n = 331). Among these cases, four cases of breast AC and two cases of cutaneous AC were identified and defined as the ORIEN apocrine subset. The remaining breast and skin cancer cases were defined as the ORIEN non-apocrine subset and were used as the training cohort for transcriptomic score development. De-identified clinical, pathological, and sequencing data, including RNA sequencing and whole-exome sequencing (WES) data, were obtained from the ORIEN Avatar program under Institutional Review Board-approved protocols at Roswell Park Comprehensive Cancer Center (approved number: BDR 198425). The YCU cohort consisted of patients diagnosed with breast AC (n = 4) or primary cutaneous AC (n = 2) at the departments of breast surgery and dermatology, YCU hospital, between 2017 and 2023. These cases were defined as the YCU reference subset. In addition, ACs arising in the axillary region with unknown primary origin (breast versus cutaneous) were included and defined as the YCU unknown-primary subset (n = 4). The study was approved by the IRB of YCU (approved number: F240200004). Clinical and pathological data were collected retrospectively from electronic medical records. RNA was extracted from formalin-fixed paraffin-embedded (FFPE) core needle biopsy specimens obtained at diagnosis or from residual FFPE tissue from surgical resections.

Publicly available transcriptomic datasets were accessed for additional analyses. Transcriptomic and clinical data for breast cancer and skin cancer from The Cancer Genome Atlas (TCGA) [25], the Cancer Cell Line Encyclopedia (CCLE) [26] and MET500 [27] were downloaded from cBioPortal and UCSC Xena and analyzed. All datasets were accessed and downloaded in October 2025.

### Sequencing methods

For the YCU cohort, FFPE specimens were sectioned at 10 um thickness and stained with hematoxylin and eosin (H&E). Tumor regions were selectively isolated using laser microdissection (LMD7000, Leica Microsystems). Total RNA was extracted using the RNeasy FFPE Kit (Qiagen, Venlo, Netherlands) according to the manufacturer’s instructions. RNA sequencing was performed by RIKEN Genesis Co., Ltd. For the ORIEN cohort, transcriptome and WES data were generated through the ORIEN Avatar program using the standardized Total Cancer Care (TCC) protocol as previously described [28].

### Transcriptomic analysis

Principal component analysis (PCA) was conducted using the gene expression values to visualize global transcriptomic differences between breast and cutaneous ACs. Differentially expressed gene (DEG) analysis was performed using the limma package in R. To evaluate pathway activity at the individual sample level, single-sample gene set enrichment analysis (ssGSEA) scores were calculated using curated gene sets from the Molecular Signatures Database (MSigDB) [29] using fgsea package. These scores represent the relative enrichment of predefined biological pathways in each sample. The relative abundance of immune cell populations in the tumor microenvironment was estimated using the xCell algorithm[30]. Cytolytic activity (CYT) scores were calculated as the geometric mean of granzyme A (GZMA) and perforin (PRF1) expression as described by Rooney et al. [31] Gene expression levels of selected individual genes were analyzed to evaluate the expression patterns of hormone receptor–related and other biologically relevant markers. PAM50 intrinsic subtypes were inferred using the genefu package[32] in R.

### Development of a transcriptomic score to distinguish breast and cutaneous tumors

Because ACs are extremely rare and the available sample size was limited, a transcriptomic classification model to distinguish breast and cutaneous origins was developed using a larger cohort of non-apocrine breast and skin cancers from the ORIEN cohort non-apocrine subset as the training cohort. The model generated a transcriptomic score, termed the BAC score. Genes with low variability were filtered based on the median absolute deviation (MAD), retaining genes above the 25th percentile of MAD calculated within the training samples. Gene expression values were standardized using Z-score normalization based on the mean and standard deviation calculated from the training cohort. To incorporate biologically informative genes, differential expression analysis between breast and skin tumors in the training cohort was performed using the limma package, and the top differentially expressed genes were assigned reduced penalty weights (penalty factor = 0.5) in the model to encourage their selection. A LASSO logistic regression model was then fitted to predict breast versus skin origin. Class weights were applied to account for imbalance in sample sizes between the two tumor types. The regularization parameter (λ) was selected using 10-fold cross-validation based on the one-standard-error rule (λ_1_se). The resulting model generated a transcriptomic score (BAC score), defined as a linear predictor:

$$\text{S}=\beta_{0}+\sum \;\beta_{\text{i}}\;{\text{X}}_{\text{i}}$$

where x_i_ represents the standardized expression level of gene i and β_i_ represents the corresponding regression coefficient.

The BAC score was developed by leveraging multiple independent breast and skin cancer cohorts (TCGA, breast n = 1081, cutaneous n = 470; CCLE, breast n = 77, cutaneous n = 119; MET500, breast n = 159, cutaneous n = 139), as well as the ORIEN apocrine subset (breast AC, n = 4; cutaneous AC, n = 2) and the YCU reference subset (breast AC, n = 4; cutaneous AC, n = 2). Within the YCU reference subset, the optimal cutoff value for the BAC score was determined using the Youden index. This cutoff was subsequently applied to the YCU unknown-primary subset to assign predicted labels. Transcriptomic features of these predicted cases were then compared with those of the YCU reference subset.

## Results

### Morphological and clinicopathological characteristics of breast and cutaneous AC

**Figure 1:**
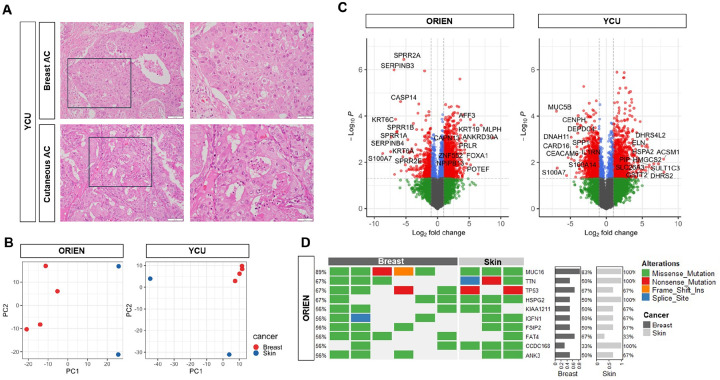
Overview of transcriptomic and mutational features of breast and cutaneous ACs in the ORIEN and YCU cohorts. (A) Representative hematoxylin and eosin (H&E) images of breast and cutaneous ACs in the YCU cohort. Left panels show low-power views and Right panels show corresponding high-power views of the same cases. Scale bars are shown in the figure. (B) Principal component analysis (PCA) of transcriptomic profiles in the ORIEN and YCU cohorts. Each point represents one tumor sample; red indicates breast AC and blue indicates cutaneous AC. (C) Volcano plots of differential gene expression between breast and cutaneous ACs in the ORIEN and YCU cohorts. The top 10 upregulated and top 10 downregulated differentially expressed genes are labeled. Red points represent significantly upregulated genes, blue points represent genes that were statistically significant but showed relatively small fold changes, and green points represent genes that did not meet the significance threshold. (D) Oncoplot of recurrent somatic alterations in breast and cutaneous ACs in the ORIEN cohort. Each column represents a tumor sample and each row represents a gene. Alteration types are color-coded as indicated. Alteration frequencies for each gene are shown on the right side of the plot.

We first examined the morphological and clinicopathological features of breast and cutaneous ACs. On hematoxylin and eosin (H&E) staining, both tumor types showed highly similar morphological features (Fig. 1A), exhibiting features of apocrine differentiation, including abundant eosinophilic cytoplasm and prominent nucleoli.

The clinicopathological characteristics of breast and cutaneous ACs in the ORIEN apocrine subset (breast AC, n = 4; cutaneous AC, n = 2) and the YCU reference subset (breast AC, n = 4; cutaneous AC, n = 2) are summarized in Table 1. The median age of patients with breast AC was 61 years in the ORIEN cohort and 70 years in the YCU cohort, whereas the median age of patients with cutaneous AC was 68 and 50 years, respectively. No consistent age pattern was observed between the two tumor types across cohorts. All breast AC cases occurred in female patients, whereas cutaneous AC occurred in one female and one male patient in the ORIEN cohort, and in two male patients in the YCU cohort. The anatomical sites of cutaneous AC the scalp (n = 1) and head (n = 1) in the ORIEN cohort, and the scalp (n = 1) and neck (n = 1) in the YCU cohort. In the ORIEN cohort, ER was negative in three cases of breast ACs (75%), and HER2 was negative in three cases of breast tumors (75%). ER and HER2 status were unavailable for cutaneous tumors. In the YCU cohort, ER was negative in all tumors. HER2 was positive in one breast AC (25%) and in two cutaneous AC (50%).

### Transcriptomic and genomic overview of ACs

Transcriptomic and genomic analyses were performed for breast and cutaneous AC cases from the ORIEN and YCU cohorts. Tumor purity of RNA-seq samples was estimated using the ESTIMATE package (**Supplementary Table S1**). The median tumor purity was 0.68 (range, 0.65–0.74) in the ORIEN cohort and 0.90 (range, 0.72–0.94) in the YCU cohort, indicating overall high tumor content in both datasets. Principal component analysis (PCA) based on the top 1,000 highly variable genes demonstrated transcriptomic differences between breast and cutaneous ACs in both cohorts (Fig. 1B). In both cohorts, breast AC cases clustered closely, whereas the two cutaneous AC cases were more widely dispersed in the PCA plot, suggesting greater transcriptomic variability among cutaneous tumors.

To further investigate these transcriptomic differences, differentially expressed gene (DEG) analysis was performed between breast and cutaneous ACs in both cohorts (Fig. 1C). Volcano plots with the top 10 differentially expressed genes labeled are shown for each cohort. Genes associated with epidermal differentiation, including KRT6A (keratin 6A), SPRR1A (small proline-rich protein 1A), SERPINB3 (serpin family B member 3), and S100A7 (S100 calcium-binding protein A7), were enriched in cutaneous tumors, whereas mammary epithelial lineage markers such as FOXA1 (forkhead box A1), PRLR (prolactin receptor), and ANKRD30A (ankyrin repeat domain 30A) showed higher expression in breast ACs. In addition, several genes previously reported to be associated with apocrine differentiation were identified among the differentially expressed genes in the breast tumors of the YCU cohort, including PIP (prolactin-induced protein), ACSM1 (acyl-CoA synthetase medium-chain family member 1), and HMGCS2 (3-hydroxy-3-methylglutaryl-CoA synthase 2).

Somatic mutation analysis was performed in the ORIEN cohort (Fig. 1D). The overall mutational landscape was broadly similar between breast and cutaneous ACs, with no prominent differences observed between the two groups. The most frequently altered genes included MUC16 (mucin 16), TTN (titin), and TP53 (tumor protein p53), along with mutations in genes involved in extracellular matrix organization and cell adhesion, such as FAT4 (FAT atypical cadherin 4) and HSPG2 (heparan sulfate proteoglycan 2).

### Breast AC enriched steroid hormone response-related pathways compared with cutaneous AC

**Figure 2:**
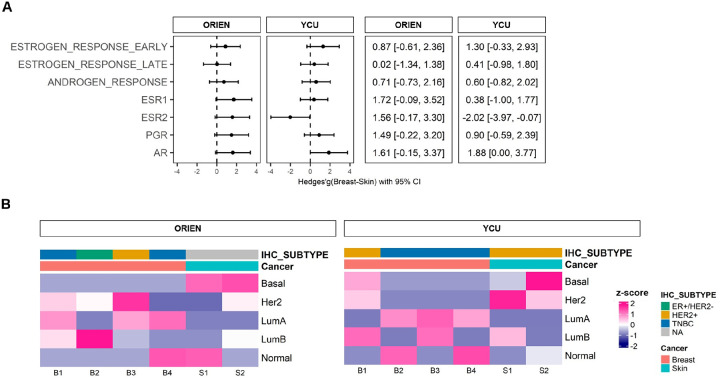
Hormone receptor–related signaling and PAM50 intrinsic subtypes in breast and cutaneous ACs. (A) Forest plot of effect size (Hedges’ g) for hormone receptor–related genes and pathways comparing breast AC and cutaneous AC in the ORIEN and YCU cohorts. Positive values indicate higher expression or pathway activity in breast ACs relative to cutaneous ACs. Points indicate effect sizes and bars indicate 95% confidence intervals. (B) Heatmaps of z-scores for PAM50 intrinsic subtype signatures across individual AC samples in the ORIEN and YCU cohorts. Columns indicate tumor samples (B1–B4: breast AC; S1–S2: cutaneous AC). Rows indicate PAM50 subtype signatures (Basal, HER2-enriched, Luminal A, Luminal B, and Normal-like). Colors indicate Z-score–normalized pathway activity. Annotation bars above the heatmaps indicate immunohistochemical subtype and tumor origin.

Next, given the known association between apocrine differentiation and steroid hormone signaling, steroid hormone response–related features were compared between breast and cutaneous ACs (Fig. 2A). Effect sizes were estimated using Hedges’ g (Breast–Skin) with 95% confidence intervals. The ssGSEA scores for estrogen response early, estrogen response late, and androgen response pathways showed positive effect sizes in both cohorts, although the 95% confidence intervals included zero. Similarly, the expression levels of ESR1, ESR2, PGR, and AR showed generally positive effect sizes, indicating a tendency toward higher steroid hormone response–related signaling in breast ACs compared with cutaneous ACs.

To further characterize the molecular phenotype of these tumors, intrinsic subtypes were inferred using the PAM50 classifier (Fig. 2B). Breast ACs tended to show higher Luminal A and Luminal B subtype scores, whereas cutaneous ACs showed relatively higher Basal-like subtype scores. Although the small sample size precludes definitive subtype assignment, these patterns suggest that breast ACs are more closely related to luminal breast cancer phenotypes, while cutaneous ACs exhibit transcriptomic features more consistent with basal-like profiles.

### Cutaneous AC enriched cell proliferation-related pathways compared with breast AC

**Figure 3:**
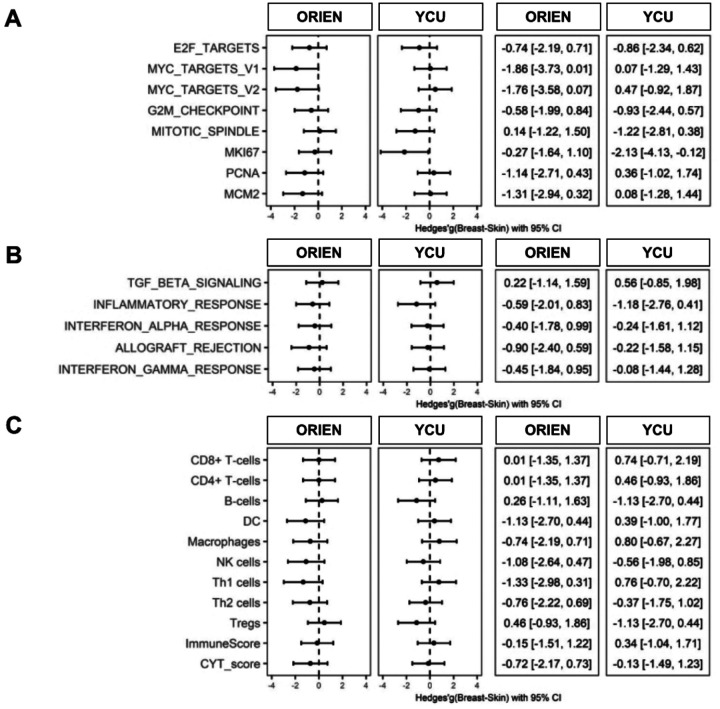
Proliferation and immune-related features in breast and cutaneous ACs. (A) Forest plot of effect sizes (Hedges’ g) for proliferation-related genes and pathways comparing breast and cutaneous AC in the ORIEN and YCU cohorts. Positive values indicate higher expression or pathway activity in breast ACs relative to cutaneous ACs, whereas negative values indicate higher activity in cutaneous ACs. Points indicate effect sizes and bars indicate 95% confidence intervals. (B) Forest plot of effect sizes (Hedges’ g) for immune-related pathways comparing breast and cutaneous AC in the ORIEN and YCU cohorts. (C) Forest plots of effect sizes (Hedges’ g) for immune cell infiltration scores and immune-related scores comparing breast and cutaneous AC in the ORIEN and YCU cohorts. Immune cell population and immune-related scores include CD8^+^ T cells, CD4^+^ T cells, B cells, dendritic cells, macrophages, NK cells, Th1 cells, Th2 cells, regulatory T cells (Tregs), the overall immune score, and CYT score. Positive values indicate higher estimated immune infiltration in breast ACs relative to cutaneous ACs.

To further assess proliferative activity, proliferation-related markers and pathways were examined (Fig. 3A). Effect sizes were defined as Breast–Skin, with positive values indicating higher expression in breast tumors. The expression of MKI67 (Ki-67), PCNA (proliferating cell nuclear antigen), and MCM2 (minichromosome maintenance complex component 2), widely used indicators of tumor proliferation, was higher in cutaneous ACs than in breast ACs, as reflected by negative effect sizes in both cohorts, with the 95% confidence interval entirely below zero in the YCU cohort. Similarly, ssGSEA scores for proliferation-related pathways—including E2F targets, MYC targets v1, MYC targets v2, G2M checkpoint, and mitotic spindle—were also higher in cutaneous ACs, with predominantly negative effect sizes (Breast–Skin). In the ORIEN cohort, all five pathways showed negative median effect sizes, whereas in the YCU cohort negative effect sizes were observed for E2F targets, G2M checkpoint, and mitotic spindle. Collectively, these findings suggest a tendency toward higher proliferative activity in cutaneous AC compared with breast AC.

#### There were no clear-cut trends in immune-related pathway activities and immune cell compositions between breast and cutaneous AC

Given the observed differences in proliferative activity, we next examined immune-related features of the tumor microenvironment. The ssGSEA scores for immune-related pathways, including inflammatory response, interferon alpha response, allograft rejection, and interferon gamma response, generally showed negative effect sizes, although the 95% confidence intervals included zero. In contrast, TGF-β signaling showed a positive effect size in both cohorts (Fig. 3B). Likewise, xCell-based estimation of immune cell composition, as well as overall immune activity metrics such as the xCell ImmuneScore and CYT score, showed no consistent differences between breast and cutaneous ACs across immune cell populations regardless of lymphoid or myeloid lineage (Fig. 3C).

### The Breast Apocrine Carcinoma (BAC) Score distinguishes between breast AC from cutaneous AC

**Figure 4:**
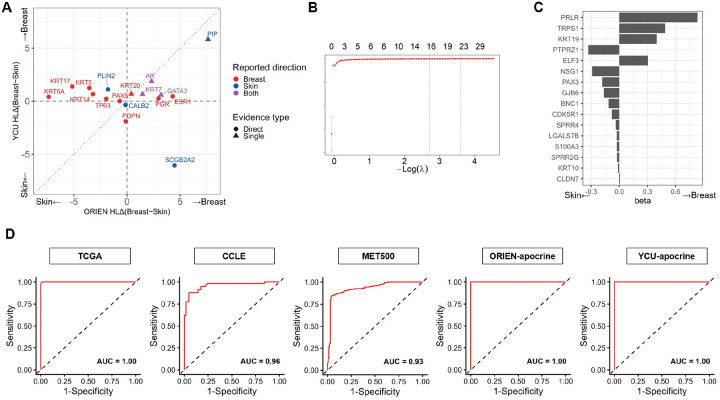
Development and validation of the BAC score for distinguishing breast and cutaneous tumors. (A) Effect sizes (Hedges’ g; Breast–Skin) for previously reported immunohistochemical markers used to distinguish breast and cutaneous ACs in the ORIEN and YCU cohorts. Genes previously reported to be positive in breast AC are shown in red, those reported to be positive in cutaneous AC are shown in blue, and those reported to be expressed in both tumor types are shown in purple. Circles indicate markers evaluated in studies directly comparing breast and cutaneous ACs, whereas triangles indicate markers evaluated in studies assessing only one tumor type. (B) Cross-validation plot of the LASSO logistic regression model used to construct the classifier. The number of non-zero coefficients at each value of the regularization parameter (λ) is shown above the plot. Vertical dashed lines indicate the λ values corresponding to the minimum cross-validation error (λ_min) and the one-standard-error rule (λ_1se). (C) Bar plot of regression coefficients of the genes selected in the final model. Positive coefficients indicate genes associated with breast tumors, whereas negative coefficients indicate genes associated with skin tumors. (D) Receiver operating characteristic (ROC) curves evaluating the performance of the BAC score in independent datasets, including TCGA, CCLE, MET500, and the apocrine subsets of the ORIEN and YCU cohorts.

Next, we explored whether transcriptomic data could be used to distinguish breast ACs from cutaneous ACs [2] [18] [33] [34] [6] [35]. First, we examined the gene expression levels of immunohistochemical (IHC) markers that have previously been reported to be useful for differentiating breast and cutaneous ACs. For each gene, the magnitude of the difference between breast and cutaneous tumors was evaluated using the Hodges–Lehmann median difference in the ORIEN and YCU cohorts (Fig. 4A). Among the examined markers, PIP, also known as GCDFP-15, which has been reported to be expressed in cutaneous apocrine carcinomas [35], was consistently more highly expressed in breast AC in both datasets. This pattern differs from previous reports describing PIP expression in cutaneous AC. No other markers showed consistent differences between cohorts in agreement with prior reports, suggesting that distinguishing these tumors based on a single marker is challenging.

This led us to generate the BAC score. Using the ORIEN non-apocrine subset as the training cohort, we constructed a predictive model to differentiate breast cancers from cutaneous cancers. The breast cancer group included tumors across all subtypes (ER-positive/HER2-negative, HER2-positive, and triple negative breast cancer), and the cutaneous cancer group included multiple tumor types such as melanoma, squamous cell carcinoma, and basal cell carcinoma. A LASSO logistic regression model incorporating differentially expressed genes was used to generate a transcriptomic score, coined the BAC score. The regularization parameter (λ) was selected using the one-standard-error rule, and 16 genes were retained in the final model (Fig. 4B, 4C). External validation was performed using independent datasets, including TCGA (breast n = 1081, cutaneous n = 470), CCLE (breast n = 77, cutaneous n = 119), and MET500 (breast n = 159, cutaneous n = 139). Across these datasets, the score accurately distinguished breast and cutaneous cancers, achieving AUCs of 1.00, 0.96, and 0.93 in TCGA, CCLE, and MET500, respectively (Fig. 4D). Importantly, when applied to AC cohorts, the score also clearly separated breast ACs from cutaneous ACs in both the ORIEN and YCU datasets.

### Application of the BAC score to the YCU “unknown-primary” subset

**Figure 5:**
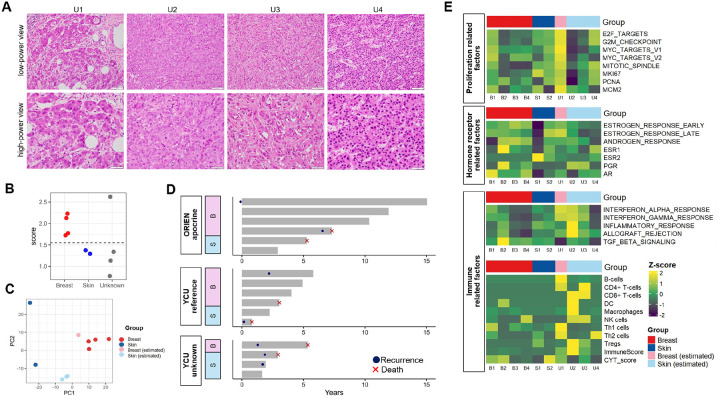
Application of the BAC score to ACs of unknown origin in the YCU cohort. (A) Representative hematoxylin and eosin (H&E) images of ACs of unknown primary in the YCU cohort. Upper panels show low-power views and lower panels show corresponding high-power views of the same cases. Scale bars are indicated in the figure.(B) Dot plot of BAC score in breast ACs, cutaneous ACs, and ACs of unknown primary in the YCU cohort. The horizontal dashed line indicates the optimal cutoff determined using the Youden index based on the YCU reference subset consisting of breast and cutaneous ACs. (C) PCA of transcriptomic profiles in the YCU cohort. Samples are colored by tumor type or estimated origin based on the BAC score, including breast AC, cutaneous AC, and estimated breast- or skin-origin tumors among the unknown-primary cases. (D) Swimmer plots of clinical outcomes of patients in the ORIEN cohort, the YCU reference subset, and the YCU unknown-primary subset. Each bar represents an individual patient, and the horizontal axis indicates time to event in years. Blue circles indicate recurrence events and red crosses indicate death. In the YCU unknown-primary subset, cases are labeled according to the predicted tumor origin based on the BAC score(B, predicted breast-origin tumors; S, predicted skin-origin tumors). (E) Heatmaps of Z-score of biological pathway activities and immune cell infiltration signatures across samples. Rows represent pathway or gene related to proliferation, hormone receptor signaling, and immune cell populations, and columns represent individual tumor samples. Sample annotations indicating tumor origin or estimated origin are shown in the bars above the heatmaps.

Finally, the BAC score was applied to the YCU “unknown-primary” subset to estimate the origin of the tumors and to examine their molecular characteristics. Representative histological images of these four cases are shown in Fig. 5A; all tumors exhibited features consistent with apocrine morphology, but the site of origin could not be determined based on morphology alone. We then used the BAC score to infer the tumor origin of these cases. Using the YCU reference subset, the optimal cutoff for the BAC score was determined using the Youden index (score = 1.55) (Fig. 5B). When this cutoff was applied to the YCU axillary tumor subset with unknown origin tumors, one tumor showed a score above the cutoff and was estimated to be of breast origin, whereas three cases showed lower scores and were estimated to be of cutaneous origin. When these four cases were visualized together with the reference subset in a PCA plot (Fig. 5C), the case estimated to be of breast origin clustered with the reference breast AC cases. In contrast, the three cases estimated to be of cutaneous origin were separated from the breast cluster but did not form a distinct cluster with the reference cutaneous tumors, suggesting greater transcriptomic heterogeneity among cutaneous AC cases.

To further elucidate their biological characteristics, clinical outcomes and molecular features were examined in the unknown-origin subset together with the reference subset. In the swimmer plot (Fig. 5D), the clinical courses of these cases showed variable patterns without a clear association with the estimated origin. In the heatmap of molecular signatures, the case estimated to be of breast origin showed relatively higher proliferation-related scores compared with the cases predicted to be of cutaneous origin (Fig. 5E). However, this pattern did not fully resemble the molecular features observed in reference breast ACs. In addition, no clear differences were observed in immune-related or steroid hormone–related signatures between the estimated groups, which may be due to extremely small number of cases.

## Discussion

In this study, we demonstrated that breast AC exhibits molecular features distinct from cutaneous AC. Across two independent cohorts, breast ACs were characterized by higher steroid hormone response–related signaling, lower cell proliferative activity, but no trend in immune-related pathway activity compared with cutaneous ACs. Also, using large cohorts of breast and skin cancers, we further developed the BAC score to distinguish tumors of breast and cutaneous origin. This score accurately differentiated non-apocrine breast and skin cancers, and importantly, it also successfully distinguished breast ACs from cutaneous ACs. When applied to axillary tumors with unknown origin, the BAC score clearly stratified cases into high-score and low-score groups, suggesting that biologically distinct entities may exist within ACs of uncertain origin. These findings suggest that, despite sharing apocrine differentiation, breast and cutaneous ACs retain distinct tissue of origin-specific transcriptional programs.

Breast and cutaneous ACs are both rare tumors [2, 12]; however, because their histopathological features are highly similar, distinguishing between these two entities particularly in the axilla has long been a clinical challenge [18]. Diagnostic difficulty particularly arises when AC develops in the axilla, where both cutaneous and breast ACs may occur.[2, 19]^,^[20]^,^[21]. In addition, even in cutaneous lesions outside the axilla, the differential diagnosis between primary cutaneous AC and cutaneous metastasis of breast cancer may be problematic [33, 36, 37]. Despite sharing a common embryological origin and close biological relationship[4–6], cutaneous AC and breast AC have been reported to exhibit distinct clinical characteristics[18]. Cutaneous AC has been reported to show relatively aggressive clinical behavior and poor prognosis[3]. In particular, the reported median survival for cutaneous AC is approximately 55 months in patients without lymph node metastasis and decreases to about 33 months in those with nodal involvement[38]. However, studies addressing the biological mechanisms underlying tumor aggressiveness or proliferative activity in cutaneous AC are largely lacking. In contrast, breast AC has been described as a biologically distinct subtype of breast cancer characterized by relatively low proliferative activity [39] and limited responsiveness to conventional chemotherapy [40]^,^[41, 42], while generally exhibiting favorable clinical outcomes, particularly within triple-negative breast cancer [13],[14], [15], which is typically associated with a poorer prognosis.. Specifically, a population-based analysis using the SEER database reported a 5-year overall survival of approximately 82% for triple-negative AC, compared with about 74% for conventional triple-negative breast cancer [15]. In line with these findings, the present study demonstrated that expression of the proliferation marker MKI67 and proliferation-related pathways tended to be higher in cutaneous ACs compared to breast ACs. Differences were also observed in hormone receptor response–related features. In previous studies, approximately half of cutaneous ACs have been reported to show ER expression [18],[43],[44], which has historically complicated the immunohistochemical distinction from breast AC. Furthermore, AR, which is closely associated with apocrine differentiation [45], has frequently been reported to be expressed in both cutaneous and breast ACs [18],[46]. In this study, however, expression of hormone receptor–related genes, including ESR1, PGR, and AR, as well as activity of steroid hormone–related pathways, tended to be higher in breast ACs. These findings suggest that, despite sharing apocrine differentiation, breast and cutaneous ACs exhibit distinct transcriptional programs that reflect their tissue of origin.

Despite these biological differences, reliable markers for distinguishing breast AC from cutaneous AC have not yet been established. Many markers currently used in clinical practice as breast cancer markers, including GATA-3, GCDFP-15, and mammaglobin target proteins associated with mammary epithelial differentiation [47]^,^[48]. However, these markers are also frequently expressed in cells with apocrine differentiation including apocrine sweat glands [49]^,^[50], limiting their utility in distinguishing breast ACs from cutaneous ACs. Several previous studies focusing specifically on ACs have proposed potential markers for distinguishing breast and cutaneous ACs, including mammaglobin, adipophilin, podoplanin, p63 and CK5/6 [2]^,^[18]^,^[33]^,^[34]^,^[6]^,^[35]. However, due to the rarity of AC, such studies remain limited, and the reported results are often inconsistent across cohorts. In the present study, the expression of previously reported markers was evaluated at the transcriptomic level in two independent AC cohorts. However, none of these genes showed expression differences that were both consistent across cohorts and concordant with previous reports.

To address this limitation, we constructed a transcriptomic classification model, BAC score, based on large cohorts of breast and skin cancers. The score incorporated genes reflecting lineage-specific transcriptional programs. Several genes associated with mammary epithelial biology, such as PRLR, TRPS1, and KRT19, were included, while genes related to cutaneous epithelial differentiation, including KRT10, SPRR family genes, and S100A3, were also represented. Importantly, commonly reported immunohistochemical markers used for distinguishing breast and cutaneous tumors were not included among the selected genes, suggesting that the classifier captured broader tissue-of-origin-specific transcriptional differences rather than relying on previously proposed single diagnostic markers. Notably, this score distinguished breast cancers from skin cancers with high accuracy in independent external datasets, including the CCLE cell-line cohort and the MET500 metastatic tumor cohort. These findings suggest that the score reflects intrinsic biological differences between breast and skin tumors rather than merely differences in the transcriptomic background of surrounding tissues. Furthermore, the score also successfully distinguished breast AC from cutaneous AC, a rare subset of tumors within these larger disease categories. Although the validation cohort was small, these results suggest the potential utility of this transcriptomic approach for the differential diagnosis of ACs.

This study has several limitations. First, the sample size of ACs was limited due to the rarity of the disease, and therefore statistical significance could not be robustly assessed for some analyses. Second, as this was a retrospective cohort study, systematic exploration of novel immunohistochemical markers for distinguishing breast and cutaneous ACs was not performed. Third, the true tissue of origin could not be definitively confirmed for the unknown-primary cases, and thus analyses involving these cases should be interpreted as exploratory. In addition, the BAC score is based on transcriptomic data, and further work will be required to translate this approach into routine clinical diagnostics. Future studies with larger cohorts will be necessary to validate these findings and further clarify the biological differences between breast and cutaneous ACs.

In conclusion, this study provides a comprehensive biological characterization of breast and cutaneous ACs. Breast ACs were associated with higher steroid hormone response–related signaling, lower proliferative activity, but no trend in immune-related pathway activity compared with cutaneous ACs. Taken together, these factors indicate distinct biological features between the two tumor types. In addition, the BAC score accurately distinguished tumors of breast and cutaneous origin not only across breast cancers and skin cancers as a whole, but also within AC cohorts. Application of this score to ACs of unknown primary further suggested its potential utility for aiding differential diagnosis in clinically challenging cases. Together, these findings highlight the potential value of transcriptomic profiling for improving the biological classification and diagnostic evaluation of ACs.

## Supplementary Material

Supplementary Files

This is a list of supplementary files associated with this preprint. Click to download.

- ApocrinesupplementaryfinaltableS1.pdf

## Figures and Tables

**Table 1:** Clinicopathological characteristics of breast and cutaneous ACs in the ORIEN and YCU cohorts.

Variable	ORIEN			YCU		
	Overall	Breast	Skin	Overall	Breast	Skin
	(n = 6^1^)	(n = 4^1^)	(n = 2^1^)	(n = 6^1^)	(n = 4^1^)	(n = 2^1^)
**Age**	65 [32–74]	61 [32–74]	68 [65–70]	66 [36–76]	70 [57–76]	50 [36–64]
**Sex**						
Female	5 (83%)	4 (100%)	1 (50%)	4 (67%)	4 (100%)	0 (0%)
Male	1 (17%)	0 (0%)	1 (50%)	2 (33%)	0 (0%)	2 (100%)
**Site**						
Breast	4 (67%)	4 (100%)	0 (0%)	4 (67%)	4 (100%)	0 (0%)
Face	1 (17%)	0 (0%)	1 (50%)	0 (0%)	0 (0%)	0 (0%)
scalp	1 (17%)	0 (0%)	1 (50%)	1 (17%)	0 (0%)	1 (50%)
neck	0 (0%)	0 (0%)	0 (0%)	1 (17%)	0 (0%)	1 (50%)
**T_stage**						
pT1	1 (20%)	1 (33%)	0 (0%)	2 (33%)	2 (50%)	0 (0%)
pT2	3 (60%)	2 (67%)	1 (50%)	4 (67%)	2 (50%)	2 (100%)
pT3	1 (20%)	0 (0%)	1 (50%)	0 (0%)	0 (0%)	0 (0%)
Unknown	1	1	0			
**N_stage**						
pN0	2 (50%)	1 (50%)	1 (50%)	4 (67%)	3 (75%)	1 (50%)
pN1	1 (25%)	1 (50%)	0 (0%)	1 (17%)	1 (25%)	0 (0%)
pN2	0 (0%)	0 (0%)	0 (0%)	0 (0%)	0 (0%)	0 (0%)
pN3	0 (0%)	0 (0%)	0 (0%)	1 (17%)	0 (0%)	1 (50%)
pNX	1 (25%)	0 (0%)	1 (50%)			
Unknown	2	2	0			
**ER**						
Negative	3 (75%)	3 (75%)	0 (NA)	6 (100%)	4 (100%)	2 (100%)
Positive	1 (25%)	1 (25%)	0 (NA)			
Unknown	2	0	2			
**PgR**						
Negative	4 (100%)	4 (100%)	0 (NA)	6 (100%)	4 (100%)	2 (100%)
Unknown	2	0	2			
**HER2**						
Negative	3 (75%)	3 (75%)	0 (NA)	0 (0%)	0 (0%)	0 (0%)
Positive	1 (25%)	1 (25%)	0 (NA)	3 (50%)	1 (25%)	2 (100%)
Unknown	2	0	2			

Clinicopathological characteristics of breast and cutaneous ACs in the ORIEN and YCU cohorts.

1 Data are presented as median [range] or number (%).

## Data Availability

The datasets generated during the current study are available from the corresponding author upon reasonable request and subject to institutional regulations. TCGA, CCLE and MET500 datasets are publicly available from cBioPortal (https://www.cbioportal.org/) and the UCSC Xena platform (https://xenabrowser.net/).

## Code availability

The code used for the analyses in this study is available from the corresponding author upon reasonable request.

## Abbreviations

AC: Apocrine carcinoma
AR: Androgen receptor
BAC: Breast Apocrine Carcinoma
CCLE: Cancer Cell Line Encyclopedia
CYT: Cytolytic activity
DEG: Differentially expressed gene
ER: Estrogen receptor
FFPE: Formalin-fixed paraffin-embedded
GCDFP: Gross cystic disease fluid protein
H&E: Hematoxylin and eosin
LASSO: Least absolute shrinkage and selection operator
MAD: Median absolute deviation
MET500: Metastatic Tumor Project 500
MSigDB: Molecular Signatures Database
NST: No special type
ORIEN: Oncology Research Information Exchange Network
PAM50: Prediction Analysis of Microarray 50
PCA: Principal component analysis
PGR: Progesterone receptor
PIP: Prolactin-induced protein
PRF1: Perforin 1
PRLR: Prolactin receptor
SERPINB3: Serpin family B member 3
SPRR1A: Small proline-rich protein 1A
ssGSEA: Single-sample gene set enrichment analysis
TCGA: The Cancer Genome Atlas
TCC: Total Cancer Care
TNBC: Triple-negative breast cancer
WES: Whole-exome sequencing
YCU: Yokohama City University
